# A stem cell niche dominance theorem

**DOI:** 10.1186/1752-0509-5-4

**Published:** 2011-01-08

**Authors:** Olaf Wolkenhauer, Darryl K Shibata, Mihajlo D Mesarović

**Affiliations:** 1 Department of Systems Biology and Bioinformatics, University of Rostock, Rostock, Germany; 2 Stellenbosch Institute for Advanced Study (STIAS), Stellenbosch, South Africa; 3 Department of Pathology, University of Southern California Keck School of Medicine, Los Angeles, USA; 4 Department of Electrical Engineering and Computer Science, Case Western Reserve University, Cleveland, USA

## Abstract

**Background:**

Multilevelness is a defining characteristic of complex systems. For example, in the intestinal tissue the epithelial lining is organized into crypts that are maintained by a niche of stem cells. The behavior of the system 'as a whole' is considered to emerge from the functioning and interactions of its parts. What we are seeking here is a conceptual framework to demonstrate how the "fate" of intestinal crypts is an emergent property that inherently arises from the complex yet robust underlying biology of stem cells.

**Results:**

We establish a conceptual framework in which to formalize cross-level principles in the context of tissue organization. To this end we provide a definition for stemness, which is the propensity of a cell lineage to contribute to a tissue fate. We do not consider stemness a property of a cell but link it to the process in which a cell lineage contributes towards tissue (mal)function. We furthermore show that the only logically feasible relationship between the stemness of cell lineages and the emergent fate of their tissue, which satisfies the given criteria, is one of dominance from a particular lineage.

**Conclusions:**

The dominance theorem, conceived and proven in this paper, provides support for the concepts of niche succession and monoclonal conversion in intestinal crypts as bottom-up relations, while crypt fission is postulated to be a top-down principle.

## Background

The maintenance of a normal colonic mucosa and its transition to adenocarcinoma is an important practical problem. Such a system has multiple levels that are interdependent through a reciprocal influence of stem cells and their niche microenvironment, and between the epithelial tissue and the colon as a whole. While interdependent, these levels are also at the same time non-interacting within their respective domains of autonomy. Using the colon as an example, we present here a mathematical analysis of a cross-level principle, linking the stemness of lineages in colonic crypts to the fate of the epithelial tissue. We present a study of cross-level principles in stem cell driven tissue organization and proof that the fate of the tissue is necessarily determined by a single lineage. Our analysis, rooted in Mathematical General Systems Theory [1,2], provides a theoretical basis for the concepts of nice succession and monoclonal conversion.

The intestinal crypt serves as an example for a complex biological system in which the behavior of the whole (the tissue level) is considered to "emerge" from the functioning and interactions of the parts (the cell level). But without specifying how the emergence takes place, the concept has almost a mystical character; it is an observation rather than a contribution to understanding the phenomenon. For understanding it is necessary to identify how the tissue level relates to the cell level. Understanding such cross-level relations in complex systems is key to "demystifying" the concept of emergence. The present paper provides one example of an organizing principle that is formulated and proven as a mathematical theorem.

The adult tissue of an organism includes *stem cells *that generate cell lineages, which maintain not only the pool of stem cells but through cell division cycles also maintain and regenerate the functional tissue through differentiation and maturation. The analysis of these inherently dynamic processes is of fundamental importance for modern medicine. For example, within the intestinal crypts, the interplay between the tissue's structural and functional organization is particularly instructive. The intestinal tract is also one of the most common sites of carcinogenesis due to the mechanical and chemotoxic stress it is subjected to. The colon is organized into about 10^7 ^crypts, each of which contains about 1000 to 4000 thousand cells [3]. At the bottom of the crypt a small number of stem cells divide slowly in an environment referred to as the *niche*. The existence of a stem cell niche in colonic crypts has been demonstrated through methylation tags [4,5]. The emerging daughter cells proliferate rapidly before differentiating and maturing into functional tissue cells. The cells of the crypt walls migrate towards the top where they undergo apoptosis (cell death) and/or are shed into the gut lumen. Homeostasis in the colonic crypt therefore, over a period of a few days, involves the renewal of the epithelial cell layer that lines the crypt [6]. The normal functioning of a crypt (and hence of the entire colon) is driven by a small number of stem cells in a self-referential manner, i.e.: the cells not only influence their environment but also respond to cues from their environment. If a complex system, such as the crypt, is developed and maintained by a very small number of cells, any externally forced alterations or malfunctioning could compromise the fate of the entire organ and even that of the whole organism. For example, the number of stem cells, or more precisely an overproduction of stem cells, can be linked to hyperplastic tissue structures; a situation that may represent a high risk for further carcinogenic transformation [3,7].

In the intestinal crypt, there is an interaction between the crypt as a whole and the stem cells, between stem cells of the niche and their surrounding tissue. These relations emerge from an intricate combination of several dynamic processes through which cells divide, differentiate and mature. A whole-part relationship thus relates the lower level of stem cell divisions (and the lineages emerging thereof) with the higher level of the tissue (and emerging properties such as homeostasis or dysplasia). Although the tissue may appear stable with respect to total cell numbers and types, the underlying parts or cell lineages may in fact be quite dynamic or unstable in order to maintain macroscopic homeostasis. What we will be focusing on here is the relation between the stem cells lineages (their stemness) and the future development (fate) of the tissue. In particular, we shall study the balance of cell divisions that maintain, reduce, or expand the pool of those cells that can generate lineages (referred to as 'stem cells'), transient cells and cells committed to differentiation and maturation to maintain the normal functioning of the tissue.

At every level, from DNA replication to tissue regeneration, living systems have developed incredibly sophisticated protection mechanisms to ensure a healthy functioning of the body, which is astoundingly robust against external perturbations and injury. Central to maintenance, renewal and repair is the concept of stem cells. These are most of the time assumed to asymmetrically divide into a new stem cell and a non-stem cell daughter. This situation is stable, but not robust because there is no mechanism to compensate for accidental death of the original stem cell. For deviations from steady state, stem cells can also divide symmetrically, either to produce two stem cell daughters (to compensate for accidental death) or two differentiated daughters (to compensate for accidental expansion). The ability of stem cells to divide both asymmetrically and symmetrically allows for robust tissue homeostasis because the crypt will always approximately have the "right" number of stem cells. In light of the robustness of a healthy tissue with multiple stem cells per crypt, could it be plausible that a single stem cell and its lineage becomes dominant for the (mal)functioning of a tissue? The astounding result of our analysis is - yes - a single lineage dominates the fate of the tissue, not as a random or rare event but as the only logically feasible outcome of a robust system.

### Processes of dominance in colorectal cancer

Gastrointestinal stem cells and their environment have the capacity to give rise to epithelial cell lineages through a regulation of different types and rates of cell divisions. This phenomenon is accompanied by mechanisms that allow for the regulation of cell differentiation and apoptosis, and that consequently ensure tissue homeostasis. However, stem cells are also key elements in the earliest stages of gastric and colonic cancer, as they form a target for mutations that may eventually lead to the development of the malignant phenotype. Due to a lack of reliable markers, adult gastrointestinal stem cells are difficult to define and characterize at the molecular level. This limits the knowledge about them and is a reason why events of early gastrointestinal carcinoma formation and expansion continue to puzzle us.

The "unitarian hypothesis" of Cheng and Leblond [8] was one of the first arguments for the dominance of single cells in the context of intestinal cancer. They suggested that all of the differentiated cell lineages within the intestinal epithelium are derived from a single stem cell lineage. Although this idea has been contested on the basis that there is no experimental confirmation [9], the idea that all the gastrointestinal epithelial cells emanate from a single progenitor stem cell is widely accepted. With the exception of the villous epithelium in the small intestine, in which the tissue a mixture derived from stem cell lineages of more than one crypt, all epithelial cell lineages within a single crypt in the small intestine and colon are clonally derived. Turning our attention from healthy functioning tissue to its malfunction, a well-established and generally accepted model of tumor progression in the colon is the adenoma-carcinoma sequence. In this framework, the morphological changes progress from aberrant crypt foci (ACF), through adenoma, and finally to carcinoma. The earliest recognizable lesions are the ACFs, which are probably monoclonal pre-neoplastic lesions involved in colon tumorigenesis in both humans and rodents (see [7] and references therein). Morphologically, these lesions appear as crypts with a thickened epithelium elevated above the mucosa and while the non-dysplastic type shows little genetic change, ACFs with associated dysplasia contain important genetic lesions. The monoclonal origin of cancer has been suggested on the basis of observations according to which neoplasms generally arise from a single cell of origin [10]. Similarly, while of course more than one cell can pass the threshold for malfunction at the same time, most of a tumor can usually be identified as the progeny of a single cell, or very few cells [11]. For the hematopoietic system, Glauche *et al. *[12] developed a stochastic model of lineage specification as a progressive restriction of lineage potential due to a competition between different interacting lineage propensities. The competition is governed by environmental stimuli promoting a drift from a multipotent coexpression to the dominance of one lineage. A review of the literature leads us to consider three processes that are examples of "dominance" by individual cells and their lineages.

*Niche succession *is a process of "dominance" by which the progeny of a single stem cell replace other stem cells in the niche [13]. Recent studies using methylation changes as stem cell fate markers revealed that niche succession appears to occur in human colonic crypts [5]. Dominance or succession occurs with symmetrical divisions where both daughter cells adopt the same fate. With symmetric stem cell division, a lineage may become extinct if both daughter cells leave the niche. This extinction can be compensated by another symmetric division in which both daughter cells remain as stem cells in the niche, resulting in no net change in stem cell numbers. Eventually all stem cell lineages within a crypt except for one become extinct, leading to niche succession or *monoclonal conversion *[14]. This dominance process recurs such that niche succession occurs multiple times during a lifetime [13]. Niche succession is a way by which a single stem cell line can expand to dominate a single crypt, while *crypt fission *is the process by which progeny can expand laterally by crypt duplication. In humans, mucosal growth involves the reduplication of crypts by crypt fission. Likewise, after mucosal damage in ulcerative colitis, crypt fission is an important regenerative mechanism. The concept of crypt fission is supported by clonality experiments in both mice and humans (see [15] and references therein). Through a model of clonal evolution in the intestinal crypt, which was, based on a simultaneous activity of several coexisting tissue stem cells, and that generate several clones at any time (thus demonstrated polyclonality), Loeffler and Roeder [16] showed how fluctuations in the long run prevent coexistence and, lead to monoclonality. Subsequently, the descendents from one clone will eventually generate all active stem cells in the crypt tissue.

The (sub)system of colonic crypts consists of two levels - stem cells and the tissue generated from their lineages. The dominance theorem, introduced and proven in this paper, establishes monoclonal conversion and niche succession as bottom-up cross-level relations. Crypt fission will be postulated to realize a top-down process.

### A stemness process model

**Figure 1:**
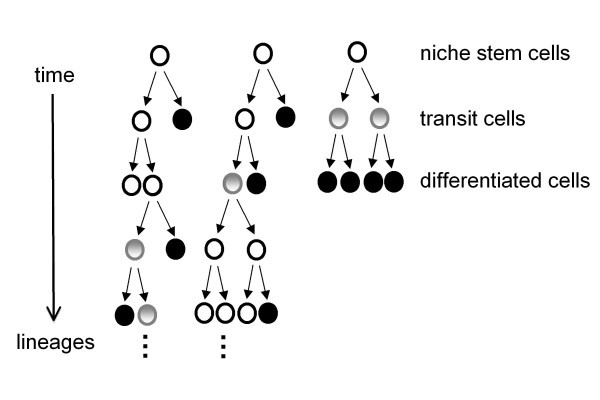
**Stem cell model**. Without loss of generality to the result of our analysis, we assume a simple stem cell model with three kinds of cells and where stem and transient cells can divide symmetrically and asymetrically. Through cell division lineages are generated. The stemness of the lineages is assessed with respect to their capacity (propensity) to contribute towards a specific future development (fate) of the tissue. A lineage that generates a long and wide tree would have a much higher stemness than a thin and short branch.

In Potten and Loeffler's [3] "screw-model" for adult tissue stem cells, proliferation and differentiation/maturation are independent processes and we furthermore allow for de-differentiation of transit cells. In order to illustrate our analysis, but without loss of generality to the results, we simplify the picture by distinguishing only three kinds of cells (Figure 1): *Stem cells*, from which lineages originate, and that are capable of generating a succession of stem cells, transit cells and mature cells; *Transit cells*, which have not yet reached their full functional competence and which can potentially de-differentiate and proliferate; and *Mature cells *that are committed to differentiation and/or have matured into functional tissue cells. In our example, we shall distinguish between three *tissue fates*: (Table 1).

**Table 1:** Tissue Fates

*Tissue Homeostasis*:	Healthy and normal functioning of the tissue.
*Clonal Expansion*:	In comparison to homeostasis, an increase in the number of proliferating cells, leading to aberrant/dysplastic tissue.
*Niche Depletion*:	A reduction of stem cells, leading to decline in the capacity for tissue renewal.

Human aging is linked to an overall decline in tissue regenerative potential, thus pointing to adult stem cell functionality [17-19]. While it remains an open question how stem cells lose their functionality over time, and despite suggestions that the lifespan of any species is not determined by a limited supply of its stem cell population [20], it does not seem implausible to link niche depletion with aging. For the present paper, we shall link clonal expansion to aberrant tissue (*dysplasia*, [21]) as a predecessor to tumor formation [22,23]. We do note however that this particular choice and simplified picture of tissue fates does not affect the generality of the formal analysis developed further below. In our formal analysis we made an effort to ensure that the results do not depend on this particular choice.

By 'stem cell', we mean a cell that is the starting point for a lineage and we shall put more emphasis on the process of lineage formation (respectively extinction) than on the stem cells. In fact, we feel increasingly uncomfortable with the notion of 'a' stem cell and instead prefer to speak of the stemness of a lineage, defined by the lineages' potential or capacity to contribute to a tissue fate. In the following section, we define stemness therefore not as a property of a cell but as an emergent phenomenon of the lineage that generates, maintains and regenerates a tissue. This point has also been argued by Lander [24] recently. That is, in the context of tissue organization, the concepts of self-renewal and potency have long been seen as key characteristic features of stem cells but all attempts to link these concepts to molecular characteristics of the cell have, so far, been unsuccessful. Our analysis demonstrates the usefulness of a definition of stemness as a property linked to a process with emergent properties [16,25].

Our mathematical framework relies on order relations, specifically adopting ideas from consensus theory [26], and is thus strikingly simple compared to the sophisticated mathematical models that have been developed to simulate intestinal crypts. This type of analysis is not restricted by the example chosen, or sensitive to the precise assumptions made about possible cell or tissue fates. In other words, the results should apply to any number of stem cells (greater or equal than two) and should be valid even if many aspects of crypt biology are uncertain, as long as stem cells have three or more alternative fates. Furthermore, we require that the results of the analysis should not be sensitive to alternative sets of potential fates ("regularity"), nor should it depend on changes to the set of actually considered fates ("consistency").

We formulate a theorem which shows that if dominance of a single cell lineage is not permitted, then there exist no other logically feasible explanation for how individual cell fates are aggregated into the collective fate of the tissue. This result can be formulated in another way, stating that dominance of a single stem cell lineage is the only outcome which satisfies the theorem, given requirements and conditions set out before. Many intuitively appealing rules, like the majority principle, fail to provide a rational explanation. The analysis supports experimental evidence for the dominance of single stem cells and their progeny in tissue maintenance and carcinogenesis. We also sketch a proof for the dominance theorem and provide intuitive graphical illustrations to support the mathematical analysis. Full details of the mathematical framework and proof are provided in the Methods section. Finally, we discuss the interpretation of the result and its consequences.

## Results

### A definition of stemness

There is little doubt that stem cell lineages are uniquely capable of developing, maintaining and regenerating tissue, and yet the concept of stem cells itself still causes confusion and concern [3,16]. In an attempt to resolve some difficulties associated with this concept, Mikkers and Frisen [27] described the development of a cell along a certain lineage as a linear process with a gradual specialization through determination, commitment and final differentiation. Besides, most cells in the mature body are at the very end of the differentiation line, while some cells - stem cells - halt the differentiation process, divide and can give rise to more cells of their own type. In this view, stem cells differ from transit amplifying progenitor cells, which may be multipotent and are capable of self-renewal, but are not halted at an intermediate position, hence inevitably progressing towards terminal differentiation.

Following Lander [24], we consider *stemness *an emergent property of cell lineages within their specific environment. Properties or attributes of stem cells can only be assessed in terms of the cells' future potential or of the retrospect, that is, by looking at the lineage they generated. More specifically, we shall define stemness as the lineage's contribution towards a tissue fate. We shall thus assume that at any point in time one can assess the lineages for their stemness. While it may not be possible to do this quantitatively, the least we can do is assess this in a qualitative manner using order relations, thus assessing the lineages' stemness with respect to:

(*h*): The capacity to produce functional, mature tissue cells

to regenerate tissue (e.g. after injury)

to maintain tissue homeostasis.

(*e*): The capacity to expand the pool of stem cells

by self-replication (symmetric cell division)

by (de)differentiation.

(*d*): The potential to reduce the pool of stem cells

by self-reduction (symmetric cell division) and maturation

by apoptosis.

Stem cells usually divide asymmetrically to produce one stem and one non-stem daughter. If this was the only choice, the system or tissue would not be robust because there would be no way to compensate for accidental death or injury. So each crypt contains at least two stem cells. But for a more robust system, stem cells, through the development of lineages, have choices - asymmetric renewal, symmetric expansion, and symmetric extinction. The notion of "choice" (inherent to fate) will lead us to a *dominance theorem*, introduced and proven further below. It demonstrates that a robust (self-adjusting) system always ends up with dominance.

Since we wish to investigate what is 'in principle' possible, we shall explore all logically feasible possibilities, and thus will not be required to consider numbers or actual order relationships between different cell lineages. Although this may sound contradictory, it is the striking simplicity of the maths involved here that will enable us to analyze very complex systems. A consequence of the kind of analysis pursued here is that it does not provide an explanation about what causal mechanisms are relevant but what logically plausible explanations, if any, are available.

**Figure 2:**
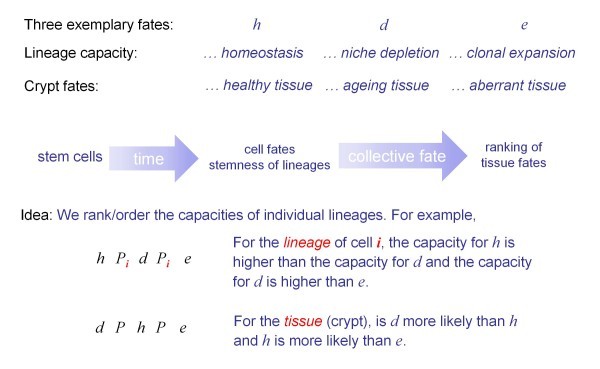
**General outline of the approach**. What we are aiming for with our analysis is an explicit description of how the fate of individual cell lineages (their stemness) is related to the fate of the tissue. By a "cross-level principle" we refer to a rule by which the stemness of lineages constituting a tissue can be related to the fate of that tissue. The stemness is assessed in terms of the capacity of the cell lineages to contribute towards a tissue fate. The mathematical implementation uses order relations. A specific instance of a cross-level principle will be encoded formally by a function, called "lineage-tissue mapping". *xP_i_y *denotes that *x *is more feasible than *y*, that is, the propensity of *x *is greater than that of *y*. The subscript *i *refers to a particular lineage, while *P *without the subscript refers to the tissue.

Figure 2 provides a graphical summary of our approach applied to the intestinal crypt. As indicated in the list above, the letters *h*, *e*, and *d *denote three attributes of stemness, which hereafter will be linked to the tissue fates (*h*omeostasis, *e*xpansion, and *d*epletion) introduced above. Because it is in practice impossible to trace lineages of stem cells and to provide a full molecular characterization, we shall here develop a number-free approach in which we rank the lineages' propensities w.r.t. tissue fates. We understand *propensity *as a disposition, natural inclination, capacity or tendency, and it should not be interpreted as relative frequency but rather as a purported cause of observed occurrences. Propensities characterize conditions that generate an effect. More specifically, at any point in time the pool of cells, their type and their lineage history encapsulates possibilities of contributions to the tissue function - possibilities that have not yet been realized, but that are nevertheless real. The propensities we attach to the possibilities can be interpreted as a measure of this status of a not yet fully realized reality - a reality in the making. The future is, in this way, present at every moment. The concept of stemness we are promoting here is thus one of a temporal allocation of regenerative potential - a property not of individual cells but of a system of lineages. We therefore support the arguments of Loeffler and Roeder [16] who suggested a definition of adult tissue stem cells not as a set of attributes of cells, but as the process by which lineages emerge in dynamically regulated process from these cells. This conceptual shift, away from the molecular characterization of a cell, towards a functional and process-oriented perspective has implications for the experiments required to study stem cells. For example, the functional definition requires the tracking of individual cells and their lineages over time [25].

### Lineage-tissue cross-level-relations

To help the reader with the formal aspects of the manuscript, Table 2 in the Methods section summarizes the notation and definitions used throughout. We formulate and analyze cross-level principles, by which the individual rankings of propensities for *n *≥ 2 lineages *L_i_*, *i *= 2,..., *n*, are aggregated into a single ranking of fates for the tissue. The ranking of propensities defines formally an order relation, where *R_i _*and *P_i _*are the propensity ranking of cell lineage *i*. For example, *h R_i _d R_i _e*, which could also be written as *R_i _*= (*h*, *d*, *e*), denotes the assessment that for the lineage of cell *i*, the propensity for *h *is greater than, or equal to, the propensity for *d*, and the propensity for *d *is greater than, or equal to the propensity for *e*. It is thus assumed that it would be - in principle - possible to assess the propensities by a "greater than" (>), respectively "greater than or equal" (≥) relation, where *hP_i_d *denotes that *h *is more feasible than *d*, that is, the propensity of *h *is greater than that of *d *w.r.t. lineage *i. hRd *denotes a "more feasible or indifferent" relation, for which the propensity of *h *is greater or equal to that of *d*. In the formal analysis the fates *h *(homeostasis), *e *(expansion), *d *(depletion) are possible values for general variables *x*, *y*, *z*. The ranking of propensities, with respect to alternative tissue fates, is then interpreted in terms of the feasibility of those fates. An instantiation of a particular cross-level principle will be referred to as a *lineage-tissue mapping*. This function maps the individual cell lineage's rankings (*lineage profiles*) into a ranking of fates for the tissue (*tissue profile*).

**Table 2:** Notation and definitions of key concepts used in the text.

*X*	The set of "potential" cell (lineage)/tissue fates.
*K*	The set of all non-empty finite subsets of *X*.

*L *= {*L*_1_, ..., *L_n_*}	The set of cell lineages.

*R_i_*	*Lineage profile*: propensities of cell lineage *i *on *X*.

(*R*_1_, ..., *R_n_*)	*n*-tuple of propensities, also referred to as a "profile".

*R*	*Tissue profile*: ranking of propensities over tissue fates - the consequence of a cross-level principle, which is determined by a lineage-tissue mapping.

*A *∈ *K*	A possible set of "actual" fates - considered in a particular context in which a lineage-tissue mapping is used to aggregate lineage profiles into one tissue profile.

*X\A*	The set of potential-but-not-actually-considered fates.

*F*	*Lineage-tissue mapping*: a map from *K *× *D *into *K *such that for all *A *∈ *K *and all (*R*_1_, ..., *R_n_*) ∈ *D*: *F*(*A*,(*R*_1_, ..., *R_n_*)) ⊆ *A*.

Ξ	Universal set of sets of alternatives (alternative fates) such that *X *∈ Ξ is one set of potential alternative fates.

Ψ	Universal set of lineages such that *L *∈ Ψ is some possible set of cell lineages.

Ω	A function that defines for a given set *X *the set *K *of non-empty finite subsets of *X*; For all *X *∈ Ξ: Ω(*X*) = *K*.

Φ	For all *X *∈ Ξ, all *L *∈ Ψ: Φ(*X*, *V*) = *D *is the set of all logically possible profiles when the set of alternatives is *X *and the set of cell lineages is *L*.

Γ	*Cross-level principle*: a map Γ defined on Ξ × Ψ such that for all *X *and *L*, Γ defines a lineage-tissue mapping the domain of which is Ω(*X*) × Φ(*X*, *L*), where Ω(*X*) = *K *and Φ(*X*, *L*) = *D *such that for any *A *∈ *K *and any (*R*_1_, ..., *R_n_*) ∈ *D*, *F*(*A*, (*R*_1_, ..., *R_n_*)) gives a ranking of propensities for tissue fates (a tissue profile).

iff	short for "if and only if".

*h, e, d*	short for "homeostasis", "expansion" and "depletion". For the tissue level these correspond to "tissue homeostasis", "aberrant tissue" (e.g. dysplasia), and "aging tissue" as a consequence of niche depletion. At the cell level they stand for the lineages' capacity to contribution towards homeostasis, clonal expansion and a reduction of stem cells.

More generally, the problem considered here is the following. Given a set of alternatives *A *and *n *rankings over *A*, we wish to identify an appropriate rule/principle that when presented with the *n*-tuple of rankings of alternatives, returns from *A *a unique consensus object that in some sense best represents the information/consequences of the individual rankings. Although the approach may seem to be abstract and technical, it is practical and concrete because it enables us to distinguish between what is logically feasible and what is not [26]. We consider *X *to be the set of *potential fates*; fates that are potentially relevant to our study. Let *K *denote the set of all non-empty finite subsets of *X*. In order to ensure that our analysis is general (law-like), we will distinguish between *X*, the set of potential fates, and a set *A *∈ *K *of *actual fates*; fates that are actually considered in a particular context. Given a set of actual(ly considered) fates *A*, the *n*-tuple (*R*_1_, ..., *R_n_*) of rankings is called a profile, where each *R_i _*is a propensity profile for the individual cell lineages *L *= {*L*_1_, ..., *L_n_*} over *A*. A cross-level principle will be encoded by a lineage-tissue mapping, merging the individual propensity rankings into a single ranked profile of propensities for the tissue. Given *X*, the set of alternative fates, which may potentially be relevant to our analysis, and the set *A *of actually considered fates for the cell lineages (at a particular point in time), the lineage-tissue mapping *F *maps elements from *K *× *D *into *K *such that for all *A *∈ *K *and all (*R*_1_, ..., *R_n_*) ∈ *D*: *F*(*A*,(*R*_1_, ..., *R_n_*)) ⊆ *A*, where with *D *we denote the set of logically possible profiles.

**Figure 3:**
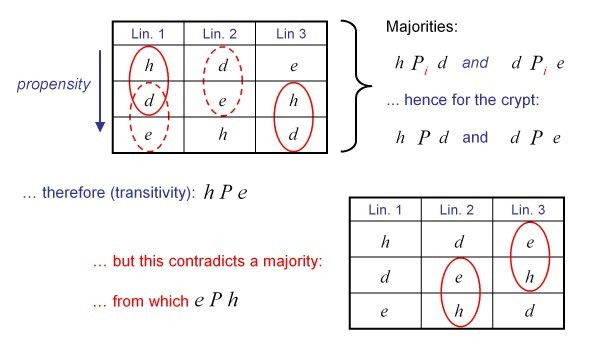
**Graphical proof for the failure of the majority principle**. The symbolic icons used to illustrate fates are summarized in Figure 2. A natural way of arriving at the collective fate would be to look for majorities of some sort. For the example in this figure, for a majority of lineages, *h(omeostasis) *is more feasible than *d(epletion) *and *d *more feasible than *e(xpansion) *- encircled in the top-left table. A rational conclusion, relying on transitivity would be to infer that therefore *h *is also more feasible than *e*. This conclusion does however contradict a majority of cell lineages for which *e *is more feasible than *h *- marked in the bottom-right table. The idea to aggregate cell fates by some sort of majority principle thus leads to inconsistencies.

An intuitive example of a cross-level principle is the *majority rule*: the fate of the tissue corresponds to the one with the greatest number of first places in the lineages propensity rankings. (We shall not consider situations in which ties occur since this has no bearing on the principle result of our analysis). Another example is *dominance*: the fate of the tissue always corresponds to the profile of a particular cell lineage. While the concept of dominance will play a greater role later, without further formal analysis one can already show that the majority rule fails to provide a contradiction-free analysis. This is illustrated in Figure 3. Rather than proceeding with various examples, and testing their validity, we seek here an analysis of what is possible "in principle". To this end, we will specify *(i) *requirements that lineage-tissue mappings should satisfy; and *(ii) *requirements which ensure that the results of our analysis are universal, general or "law-like".

With respect to the lineage-tissue mapping we identify a minimal set of requirements so as to ensure that the widest possible class of cross-level principles is covered. Specifically, we require for the lineage-tissue mappings no more than to respect *unanimity: *For all *x *and *y*, if *xR_i_y *for all lineages *L_i_*, then *xRy *for the tissue. In other words, if for every cell lineage the propensity for *x *is higher than for *y*, then for the tissue, the propensity of the tissue fate related to *x *should be greater than that of *y*.

Note that for different sets of potential fates *X*, the same rule for a cross-level principle would define a different lineage-tissue mapping. This is due to the fact that a mathematical function (or mapping) is strictly defined in combination with its domain. A cross-level principle therefore defines a lineage-tissue mapping for each set *X *of alternatives (and hence its family *K *of non-empty finite subsets) and each set *L *of cell lineages (and hence the set *D *of profiles). In what follows we will therefore distinguish between alternative sets *X*s and *A*s and discuss the consequences on lineage-tissue mappings as well as the validity of our analysis of cross-level principles in general. Changes to *X *will be dealt with in a requirement for *regularity*, while changes in *A *will be considered with a condition on *consistency*. If *X *is thus some set of potential candidate fates, whereas *A *∈ *K *is a possible set of actually considered fates, our analysis will also have to consider the elements of *X*\*A*, which are the *potential-but-not-actually-considered *fates. This is to ensure that if something is missed, this does not make any difference to the result of our analysis.

*Regularity *means, that for a given cross-level principle, a set of actually considered fates, a set of cell lineages at time *t*, and a profile (of ranked propensities) defined over them, if there exists another reduced set of potential-but-not-actually-considered fates, then in the analysis, the lineages' propensities over the remaining potential fates (including the actually considered ones) and the ranking from the set of actually considered fates should not change. In other words, we require our analysis to be independent of any other possible "universes of fates" that one may assume. Take the example above, where we consider three abstract fates - clonal expansion, homeostasis, and niche depletion. One might argue that at this level of abstraction "everything" is possible and that the approach time lacks specificity. It is for that reason that we here explicitly define requirements, like regularity, to ensure that any kind of universe of potential fates is covered by the analysis. In other words, while the definition of *X *may depend on the context and could seem to be a subjective choice, regularity ensures that the result of our analysis would not be influenced by the context or choice.

Following on from regularity, Bordes and Tideman [28] showed that regularity implies another condition referred to as *independence of irrelevant alternatives *(IIA). IIA means that the analysis of rankings among the actually considered fates should be robust in the sense that ranking of tissue fates and the lineages' propensity rankings over the actually considered fates should be the same if propensities over potential-but-not-considered candidate fates change. IIA ensures that the analysis of a subsystem of actually considered fates is not influenced by something we do not know of. In other words, if one would study a particular system and analyze the stemness of cell lineages, one can only consider as an argument to the lineage-tissue mapping of what we can have knowledge of. The Methods section gives full details related to IIA.

The situation described here is similar to that of pathway modeling in systems biology. Modeling a particular pathway, is, in most cases, the study of a *subsystem*, whose function contributes to a larger whole (e.g. signal transduction pathways linked to cell proliferation). The implicit assumption for these projects is usually that the subsystem (e.g. the wnt-pathway) can be analyzed in isolation. Although this is not often done, before conducting an analysis of a subsystem that is embedded into a larger whole, one ought to specify criteria that ensure the analysis of the subsystem will allow inferences of greater generality.

To require a cross-level principle to be regular, or a lineage-tissue mapping to satisfy IIA, means that for any fixed context, that is, any given set of actually considered fates, the ranking of tissue fates should be independent of the very existence of alternatives outside the context, that is, independent of whether potential-but-actually-not-considered fates exist. If such potential-but-not-actual fates do exist, the analysis should be independent of who they are and of what the lineages' propensity rankings over them are.

Regularity is about the possibility of alternative sets of potentially relevant cell fates, the differences of which however do not affect the set of actually considered fates. Similar, IIA is concerned with what happens to the ranking of tissue propensities when the set of actually considered fates being given, the profile of lineage propensities changes somehow. Furthermore, we add a requirement for *consistency *to our analysis to ensure that tissue fates can actually be determined for a given set of alternative lineage fates. In other words, for any two fates we require that it is possible to assess the order of propensities (greater than or indifferent), that there is some relation between the two. The condition for consistency is concerned with what happens to the ranking of tissue propensities when the profile of lineage propensities being given, the set of actually considered fates changes in a certain way. Consistency implies another condition referred to as C', and which is fully justified in the Methods section. With C' we ensure that a function exists which relates lineage propensities to a tissue fate. There is some similarity between regularity and the consistency condition (even more so with C'). In both cases, the profile of lineage propensities is assumed given, and a set of fates is reduced (and for C' the tissue fates stay the same). However, for regularity it is the set of *potential *fates that is reduced in such a way that the original set of actually considered fates is still included in the set of potential fates after the reduction. For C' the set of *actual *candidates, is reduced in such a way that the set of tissue fates is still included in the set of actually considered fates.

At this point, let us consider another intuitively appealing example for an aggregation rule, called *rank-order principle*. For each cell lineage all fates are ranked and weights (1,2,3, ...) be given in the opposite order of the ranking - the highest weighting for the fate with the largest propensity. For example, if there are three fates, the highest weighting is 3. With respect to the conceptual difference between *X *and *A*, we can proceed with our analysis in two ways [28]: The *global *interpretation of the rank-order principle would assume that it is possible to know the propensities for the whole of *X*. After weights have been assigned, the relevant tissue fates are identified and ranked by picking those that are in the subset *A *of actual fates. The tissue fate with the highest propensity corresponds to the fate with the highest weighting. The second interpretation is referred to as a *local *rank-order aggregation rule and proceeds by determining the fates' weighting from the restrictions of the lineages' propensities to a subset *A *of actual fates, the choice from *A *being the fate(s) with the highest of the weightings. Because for each given profile the global interpretation of the rank-order method generates a complete weak ordering over the whole of *X*, which will be used to determine the tissue fate over the set of actual fates, the corresponding lineage-tissue mapping satisfies the consistency condition but fails to satisfy IIA and regularity. The local interpretation of the rank-order method does satisfy regularity and IIA but fails to satisfy the consistency condition because to determine the ranking over a set of actual fates *A*, only the lineages' propensities restricted to *A *are taken into account (no information about their propensities over the potential-but-not-actually-considered fates is used). Both, the intuitive majority principle and the rank-order principle fail to provide a contradiction-free analysis: are there any other rules that could provide an explanation for cross-level relationships? The answer is given by the dominance theorem, introduced in the next section.

In summary, to ensure the most general analysis, we require the cross-level principle and the lineage-tissue mapping to satisfy requirements for regularity, IIA, consistency (including C'), and unanimity. We can now state our main result about the relationship between the stemness of cell lineages making up a tissue and the future development (fate) of that tissue.

### A theorem about cellular cross-level relationships

A cross-level relationship describes how the behavior of stem cell lineages relates to the fate of the tissue. Following on from the considerations in the previous section, we can now ask the following question: Are there any reasonable cross-level principles that satisfy our requirements? The answer is yes, but surprisingly only one, and one in which a single cell lineage is dominant. This can be formulated as a theorem:

THEOREM (dominance): *For any tissue with two or more cell lineages, where the capacities of each cell lineage are related to three or more tissue fates, the only cross-level principle (lineage-tissue mapping) that satisfies the conditions of unanimity, regularity, independence of irrelevant fates, and consistency is the dominance relationship. There are no other principles satisfying these conditions*.

The surprising result is thus that despite many intuitively appealing rules that come to mind, none of them would allow for a contradiction-free and consistent analysis.

*Sketch of a Graphical Proof for the Dominance Theorem: *A lineage is said to be dominant if the tissue profile always matches this individual lineage's profile. We define *P_i_*, *R_i _*and *I_i _*to be the "more feasible than", "more feasible or indifferent" and "indifferent" relations for lineage *L_i_*. When we write *P*, *R *or *I*, without a subscript, this indicates a relation for the tissue. Our diagrammatic proof of the dominance theorem follows the proof of Arrow's possibility theorem in [29].

**Figure 4:**
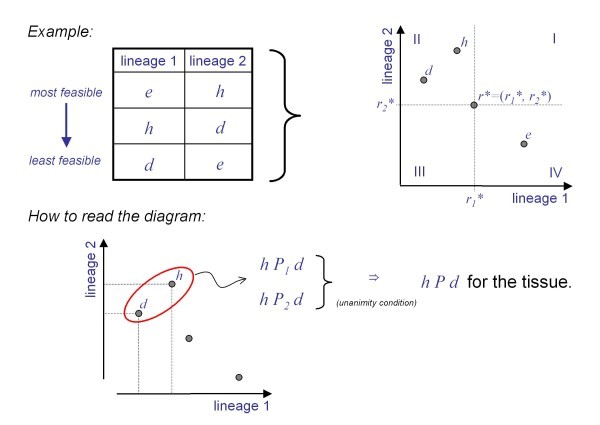
**Graphical proof of dominance**. Without loss of generality, we consider two cell lineages at time *t*, with the propensity rankings shown in the table. For the graphical presentation we translate the orderings into the Euclidean plane. Only relative positions will matter and no actual numbers are required. The proof presented here is based on Blackorby *et al. *[29].

Without loss of generality, we consider two cell lineages and use the example in Figure 4 where the propensity profiles are translated from a table into the Euclidean plane. The dots in the plane summarize the cell level propensity profiles, from which we wish to infer an order of potential tissue fates. The diagram is interpreted by comparing the positions of the points *h*, *e*, *d*, relative to each other and with respect to a reference point r*, which divides the plane into four regions. From Figure 4 we have for both lineages *hP_1_d *and *hP_2_d *and hence *unanimity*, from which we infer that for the tissue we should have *hPd*. By unanimity we can also see that all points in region I are more feasible than r* and that r* is more feasible than all points in region III.

Next we establish that all points in region II (or region IV) must be ranked in the same way against r*. That is, for all points *r *in region II, for the tissue we have either rPr*, rIr*, or r*Pr. Consider the two fates *d *and *h *and suppose for the tissue hPr*. By using an increasing monotone transformation, one could map r* into itself and *h *onto *d*, while preserving the individual rankings.

**Figure 5:**
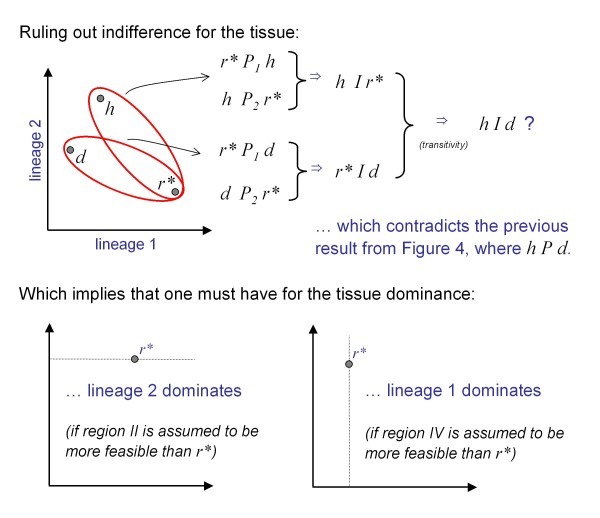
**Graphical proof of the dominance theorem**. Cont'd from Figure 4. In the upper part it is shown that indifference is not possible for the tissue order relation. The lower part shows that we can merge adjunct regions with the same ranking, which means that we end up with only two possibilities; in either case dominance is inevitable.

Since hPr*, we must therefore also have dPr* as well. In other words, the whole of region II (region IV) is ranked in the same way with respect to r* (although not w.r.t. each other). Because the lineage-tissue mapping will imply an ordering, there can be three ways by which region II (region IV) is ranked with respect to r*: indifferences, more feasible, or less feasible. Figure 5 shows that indifference can be ruled out because it leads to a contradiction; for we have hIr* and dIr*, which would imply (by transitivity) *hId *for the tissue, contradicting the unanimity inference *hPd *from Figure 4.

In the next step it is shown that the ranking given to region II must be the opposite from the ranking given to region IV. Suppose region II is more feasible than r*, one can transform *h *onto r* and r* onto *e*. Since *h *was assumed to be more feasible than r*, this relationship must be preserved by the transformation and thus r* is more feasible than *e*, and hence region IV is less feasible than r*. Note that the assumption of region II to be more feasible, is arbitrary; if region II is less feasible than r*, this would imply that region IV is more feasible.

Remembering our initial conclusions about regions I and III, we notice that we can merge adjacent that are both ranked in the same way with respect to r*. Finally, reminding ourselves that the tissue order relation implied by the lineage-tissue map, is one of *dominance *if and only if there is an individual lineage *L_i _*such that for all r* in the Euclidean Space ℝ*^n^*, if for the lineages *r*_i_* >*r_i_*, then for the tissue r*Pr. What we have shown in Figure 4 and 5 is that with respect to r*, the tissue order relation implied by the lineage-tissue mapping must lead to one of two rankings - one in which lineage 1 dominates (with assumption that region II is more feasible than r*) or one in which lineage 2 dominates (if we had assumed that region II is less feasible than r*). Dominance for the tissue is inevitable, the only plausible explanation (see Figure 5).

It is worth again emphasizing that the theorem is saying that weakly reasonable lineage-tissue mappings other than dominance simply do not exist - no need to try find one - they will never be found. For further details of a diagrammatic proof, including a demonstration that the result holds for any number of lineages, we refer to [29].

The main objective of the present paper was to investigate dominance in cross-level (whole-part) relationships of stem cell lineages and their tissue. To this end, we established a theoretical basis for the concepts of niche succession and monoclonal conversion. A key element of our approach is that we turned requirements on the cross-level principles (regularity, IIA and consistency) into requirements for the analysis, not the biophysical process, thereby ensuring the universality of our result. This gives the analysis an epistemological character - allowing a result about what we can know about the cell-tissue link, in addition to a description of what the nature of this cross-level relationship is.

## Discussion and Conclusions

The formal analysis presented here is based on a definition of stemness that is derived from an analysis of a lineage's capacity to contribute towards the future development of a tissue and hence its fate. Based on this definition we asked how one could explain the relation between stem cell lineages and tissue fates? Considering mild conditions that cross-level principles should satisfy and by posing requirements to ensure that the results are most general and that the analysis is robust, we find that dominance by an individual lineage is the only logically feasible explanation to formulate a cross-level principle. The theoretical finding does therefore confirms, or is confirmed by experimental evidence for niche succession and monoclonal conversion.

Our conceptual framework is simple and yet comprehensive when exploring all logical possibilities. The mathematical framework is quantitative (based on order relations) but the analysis is also qualitative (number-free). The level of modeling in our analysis has one consequence in that it is not just about "what is", i.e., an ontological question, but the analysis is also about "what we can know", i.e., an epistemological result. In other words, our analysis is not a *description *of causal mechanisms underlying carcinogenesis; instead the analysis demonstrates that dominance is the only logical *explanation *that can be given. In our context, this means that the goal is not so much prediction than understanding. Prediction - in the sense of knowing how the future evolves is, of course, a desirable objective - but in biology predictions are not synonymous with understanding. Generating predictions is one way to test whether this understanding is consistent with the behavior of the system of interest.

The derivation of the relation of tissue fates as an aggregation (consensus) of individual lineage propensities is without consideration for the mechanisms of how the propensities for individual stem cell lineages arise. One could describe the result as a bottom-up organization principle, formalizing an example of emergence. Just as the fate of the tissue is based on individual stem cell lineages, the latter will depend on the state of the tissue as a whole. It is this self-referential or circular causality of the tissue environment that influences the behavior of stem cells, which in turn constructs this environment that makes a detailed biophysical analysis so difficult and motivates the level and type of mathematical analysis chosen here. In complex systems, such as the intestinal tissue or a single crypt, in which multilevelness is a defining characteristic, cross-level relations are in general bi-directional, i.e., bottom-up as well as top-down. In the colon, stem cells reside within a niche, formed by epithelial and mesenchymal cells. The tissue level regulates stem cell behavior through paracrine secretion of growth factors and cytokines. While our analysis established niche succession and monoclonal conversion as bottom-up principles, the concept of crypt fission suggests itself as a natural candidate for a top-down coordination process. Clonality experiments have shown clustering of mutated, phenotypically similar crypts together in patches. Parks *et al. *[30] showed that in the small intestine and colon, administration of mutagens leads to the emergence of crypts populated by cells with a different, mutated phenotype. This is preceded by a transient rise in the frequency of crypts with a partially mutated phenotype, and the disappearance of these partially mutated crypts occurs contemporaneously with the attainment of a plateau value of the wholly mutated crypts. Crypt fission, which occurs rarely in normal colons, can explain the clustering of apparently related crypts through a process in which crypts undergo a bifurcation, eventually leading to the formation of two daughter crypts. This process is believed to play a central role in the massive increase in crypt numbers in the postnatal period and the regenerative phase following radiation ([31] and the references therein). The majority of adenomas greater than one crypt in size appear to be polyclonal [15].

The formal framework and proof requires a minimum of two stem cells and three or more alternative fates. For intestinal crypts, our analysis thus rules out a model of tissue homeostasis in which lost cells are replenished by a single dominant cell that divides *only *assymetrical. Recent, combined experimental and theoretical findings [32] suggest a stochastic picture in which stem cells can divide symmetrically, with the cell fate being determined after division, possibly by competition for available niche space at the crypt base. In this setting, homeostasis is obtained by competition of cells with respect to properties of the tissue level [33,34]. Both sets of works also showed that crypts gradually become monoclonal.

## Methods

The methods section introduces the mathematical framework, notation, definitions, and a formal proof. This section, including the full proof of the theorem, should be skipped upon first reading: The Results section includes a self-contained informal and graphical version of the proof to which the present section provides the formal background and further mathematical details.

### Order relations

A basic element of reasoning is captured by the concept of *transitivity*: If *z *is more feasible than *y *and *y *is more feasible than *x*, then *z *is more feasible than *x*. This can be defined in terms of an *order relation*, denoted *P*: If *xPy *and *yPz*, then *xPz*. We write *xPy *to mean "*x *is more feasible than *y*", that is, the propensity of *x *is greater than the propensity of *y *(w.r.t. a particular fate). If neither *xPy *nor *yPx *is true, then we say that we are "indifferent" with respect to *x *and *y *and write *xIy*, where *I *denotes the *indifference relation *and *xIy *means *xPy *and *yPx*. Finally, it is also convenient to introduce another relation, *R*, the "more feasible or indifferent" relation. This relation is also referred to as a *weak ordering *("weak" because it does not exclude indifference). This is defined as *xRy *if *xPy *or *xIy*. In other words, *xRy *unless *yPx*, or *x *is preferred to or indifferent to *y *if *y *is not preferred to *x*. The axioms on *P *being a preference relation now immediately turn into properties of *R*: (1) for all *x, y *we have *xRy *or *yRx *(i.e. *R *is anti-symmetric), and (2) for all *x, y, z*, if *xRy *and *yRz *then *xRz *(i.e. *R *is transitive). It follows then that (a) for all *x*, *xRx*, (b) If *xPy *then *xRy*, (c) for all *x, y *either *xRy *or *yPx*, (d) If *xPy *and *yRz *then *xPz*. A binary relation *R *is said to be *complete *if for all *x *and *y *in *X *it holds that *xRy *or *yRx*, or both.

### 

#### A framework for reasoning about cross-level-relations

The present text includes three versions of the proof: an informal sketch, a graphical and a formal version. The proof of the dominance theorem is inspired by Arrow's impossibility theorem in collective choice theory [35-37]. Chapters 3 and 3* of Sen's more general account [37] provide a concise proof Arrow's original "General (Im)Possibility Theorem" of 1963 - the version that is relevant here. What we take from collective choice theory is the idea of cross-level relationships, developing a formal, quantitative and yet number-free conceptual framework in which one can analyze law-like principles about what is 'in principle' possible. Like Arrow's impossibility theorem, the dominance theorem is astounding because of the relative simplicity of the analysis with which one can achieve such general, fundamental result. The use of order relations enables us to analyze a system, as complex as the intestinal crypt, in its entirety and comprehensively.

The following formal definitions are identical to Bordes and Tideman [28] as is the proof of the dominance theorem presented here is formally identical to the proof of Arrow's impossibility theorem. Because the semantics employed here for tissue organization differ from collective choice theory, we have included all details, rather than giving a reference, to make the text self-contained.

Let Ξ be some 'universal' set of sets of alternative fates such that *X *∈ Ξ is one set of potential fates. Similar, let Ψ be some 'universal' set of lineages such that *L *∈ Ψ is some possible set of cell lineages. We denote by Ω a function that defines for a given set *X *the set *K *of non-empty finite subsets of *X*. In other words, for all *X *∈ Ξ: Ω(*X*) = *K*. Furthermore, for all *X *∈ Ξ, all *L *∈ Ψ: Φ(*X*, *L*) = *D *is the set of all logically possible profiles when the set of alternatives is *X *and the set of cell lineages is *L*. We are now in a position to formalize an *cross-level principle *as the function Γ defined on Ξ × Ψ such that for all *X *and *L*, it realizes a lineage-tissue mapping, the domain of which is Ω(*X*) × Φ(*X*, *L*), where Ω(*X*) = *K *and Φ(*X*, *L*) = *D *such that for any *A *∈ *K *and any (*R*_1_, ..., *R_n_*) ∈ *D*, *F*(*A*, (*R*_1_, ..., *R_n_*)) gives a tissue profile.

We shall now look at what influence the difference between *X *and *A *can have. In particular, we shall require that our analysis does not depend on what set of potentially relevant fates, *X*, is chosen. Towards this we require the mathematical definition of a restriction: If *R *is any binary relation on a set *S*, and if *T *is a subset of *S*, *R*|*_T _*is the *restriction *of *R *to *T*.

DEFINITION (regularity). A cross-level principle Γ is *regular *iff for all (*X*, *L*) and (*Y*, *W*) in the domain of its lineage-tissue mapping, with *F *= Γ (*X*, *L*) and *G *= Γ(*Y*, *W*), if *W*=*L *and *Y *⊆ *X*, then for all *B *∈ Ω(*Y*) (and hence *B *belong to Ω(*X*) since Ω(*Y*) ⊆ Ω(*X*))) and all (*R*_1_, ..., *R_n_*) ∈ Φ(*X*, *L*),

$$F\left(B,\left(R_{1},\ldots ,R_{n}\right)\right)=G\left(B,\left(R_{1}|{}_{Y},\ldots ,R_{n}|{}_{Y}\right)\right)$$

Regularity thus means, that for a given cross-level principle, a set of actually considered fates, a set of cell lineages at time *t *and a profile (of ranked propensities) defined over them, if there exists another reduced set of potential-but-not-actually-considered fates, then in the analysis, the lineages' propensities over the remaining potential fates (including the actually considered ones) and the ranking from the set of actually considered fates should not change. In other words, we require our analysis to be independent of any other possible "universes of fates" that one may assume. Namely, while the definition of *X *may depend on the context and could seem to be a subjective choice, regularity ensures that the result of our analysis would not be influenced by the context or choice.

Following on from regularity, Bordes and Tideman [28] proved that regularity implies another condition referred to as *independence of irrelevant alternatives *(IIA):

DEFINTION (IIA). *X *and *L *being given and hence *K *= Ω(*X*) and *D *= Φ(*X*, *L*), a lineage-tissue mapping *F *satisfies *Independence of Irrelevant Alternatives *(IIA) iff for all *B *∈ *K *and all (*R*_1_, ..., *R_n_*), $\left(R_{1}^{'},\ldots ,R_{n}^{'}\right)\in D$, if for all $L_{i}\in L:R_{i}|{}_{B}=R_{i}^{'}|{}_{B}$, then

$$F\left(B,\left(R_{1},\ldots ,R_{n}\right)\right)=F\left(B,\left(R_{1}^{'},\ldots ,R_{n}^{'}\right)\right)$$

IIA means that the analysis of rankings among the actually considered fates should be robust in the sense that ranking of tissue fates and the lineages' propensity rankings over the actually considered fates should be the same if propensities over potential-but-not-considered candidate fates change. IIA ensures that the analysis of a subsystem of actually considered fates is not influenced by something we do not know of. If one would study a particular system and analyze the stemness of cell lineages, one can only consider as an argument to the lineage-tissue mapping of what we can have knowledge of.

To require a cross-level principle to be regular or a lineage-tissue mapping to satisfy IIA means that for 'any fixed context', that is, any given set of actually considered fates, the ranking of tissue fates should be independent of the very existence of alternatives outside the context, that is, independent of whether potential-but-actually-not-considered fates exist, and hence, if such potential-not-actual fates do exist, the analysis should be independent of who they are and of what the lineages' propensity rankings over them are.

While regularity is about the possibility of alternative sets of potentially relevant cell fates, the differences of which however do not affect the set of actually considered fates, we now look at another requirement that ensures our analysis is not sensitive to alternative sets of actually considered fates. Similar, IIA is concerned with what happens to the ranking of tissue propensities when the set of actually considered fates being given, the profile of lineage propensities changes somehow.

We need to add one more requirement to our analysis to ensure that tissue fates can actually be determined for a given set of alternative lineage fates. In other words, for any two fates we require that it is possible to assess the order of propensities (greater than or indifferent), that there is some relation between the two.

DEFINITION (consistency). A lineage-tissue mapping *F *with domain Ω(*X*) × Φ(*X*, *L*), taking (*K*, *D*) as an argument, satisfies the consistency condition iff for all (*R*_1_, ..., *R_n_*) ∈ *D *and all *A*, *B *∈ *K*, if *A *⊆ *B *and *A *∩ *F*(*B*, (*R*_1_, ..., *R_n_*)) ≠ Ø, then

$$F(A,(R_{1},\ldots ,R_{n}))=A\cap F(B,(R_{1},\ldots ,R_{n})).$$

The condition for consistency is concerned with what happens to the ranking of tissue propensities when the profile of lineage propensities being given, the set of actually considered fates changes in a certain way.

Bordes and Tideman [28] refer to IIA as an *interprofile *property and to consistency as an *intraprofile *property. Following them further, we state a consequence of the consistency condition above and shall denote this condition C':

$$C':\text{If}\;F(B,(R_{1},...,R_{n}))\subseteq A\subseteq A,\;\text{then}\;F(A,(R_{1},...,R_{n}))=F(B,(R_{1},...,R_{n})).$$

With C' we ensure that a function exists with which we can aggregate lineage propensities into a tissue fate.

There is some similarity between regularity and the consistency condition (even more so with C'). In both cases, the profile of lineage propensities is assumed given, and a set of fates is reduced (and for C' the tissue fates stay the same). However, for regularity it is the set of *potential *fates that is reduced in such a way that the original set of actually considered fates is still included in the set of potential fates after the reduction. For C' the set of *actual *candidates is reduced in such a way that the set of tissue fates is still included in the set of actually considered fates.

### Proof of the dominance theorem

For informal version and a graphical sketch of the proof we refer to the Results section of the present text. Because our mathematical definitions closely follow ideas from voting and collective choice theory, further material can be found the literature surrounding Kenneth Arrow's Impossibility Theorem. The rich literature related to social choice and voting theory provides various alternative demonstrations of the proof (e.g. [38]). Our presentation of the proof for the dominance theorem is analog to Sen's ([37], Chapter 3*) revision of Arrow's (1963) proof of his "General (Im)Possibility Theorem" in [36]. We strongly recommend the interested reader to consult the discussion in [28] before reading [36]. Their discussion also clarifies a number of confusing issues surrounding Arrow's original proof of the theorem. We do however insist upon Arrow's original requirement for transitive, reflexive and complete (connected) relations. While in collective choice theory one considers subjective choices, we here deal with objective, biophysical processes and what we can know about them. Whether or not we could actually quantify an order relation 'in practice' (the beauty of the proof being that this is not necessary) there is always something that can be compared 'in principle' and hence the requirement for (or assumption that there always is) a complete, transitive order relation for the tissue fates.

It is asserted that the collective fate of a tissue depends on propensities of individual stem cell lineages towards the alternative fates under consideration. We might say that the future development of the tissue (its fate) emerges from the lineages that stem cells generate. We are here concerned with the question of how the propensities of individual lineages are aggregated into a ranking of fates for the tissue.

A collective fate rule that specifies orderings for the tissue (society of cells) is called a *lineage-tissue mapping *(LTM). A LTM is a particular type of *cross-level principle *such that each ranking of tissue fates (referred to as the *tissue profile*) is a reflexive, transitive and complete order relation.

There are a couple of "reasonable" (mild) conditions that one would require from the analysis of cross-level principles. Condition of unrestricted domain: It is required that whatever principle of going from individual lineage profiles to a collective tissue profile is discussed, the LTM must be free of contradiction and inconsistencies for any logically possible set of individual orderings (lineage profiles). One would also require that any LTM must satisfy the *weak Pareto principle*, i.e., if every lineage ranks *x *over *y*, then for the tissue *x *should rank higher to *y. Independence of irrelevant alternatives*: The tissue fate over a set of alternatives must depend on the ordering of the individual lineages only over those particular alternatives, and not on anything else. Suppose the ranking is between *x *and *y*, and individual rankings of *x *and *y *remain the same, but the rankings of *x *over some other alternative *z *changes, or the rankings of *x *over another alternative *w *alters. What is required is that the tissue fate ranking between *x *and *y *should remain the same, independent of those "irrelevant alternatives". In the context of Arrow's Theorem we refer to [28] for a comprehensive discussion of this requirement. As noted before, the semantics of the analysis here differs substantially from social/collective choice and voting theory, while the mathematical reasoning is identical.

Finally, one hypothesis would be to add the requirement that a LTM should not be "dominant", that is, there should be no individual lineage such that whenever in lineage *x *ranks higher than *y*, then the tissue must rank *x *over *y*, irrespective of the rankings of other lineages. The remarkable result of the theorem, going back to Kenneth Arrow [35], is that there is no LTM that can simultaneously satisfy all these mild conditions. The result surprises because of its universal character - it is not a matter of finding a suitable LTM, there simply will never be one! Intuitive cross-level principles, such as the majority principle or the rank-order method can be shown to fail these criteria, lead to inconsistencies, or contradictions for particular examples (see Results section). For a principle to have the character of a law, it must work for all logically possible cases.

DEFINITION 1. A cross-level principle is a lineage-tissue mapping (LTM) *F*, the range of which is restricted to the set of orderings over *X*.

CONDITION 1 (unrestricted domain): The domain of the rule *F *must include all logically possible combinations of individual orderings.

CONDITION 2 (Pareto principle): For any pair, *x*, *y *in *X*, for all lineages *i*, if *xP_i_y*, then for the tissue *xPy*.

Let *xRy *represent a binary relation, specified over a set *S *such that the relation specifies a subset *R *of *S*×*S*. An element *x *in *S *is a "greatest" element of *S *with respect to a binary relation *R *if and only if (iff) for all *y*, if *x *in *S*, then *xRy*. The set of greatest elements in *S *is called *choice set*, denoted *C*(*S, R*).

CONDITION 3 (independence of irrelevant alternatives): Let *R *and *R' *be the tissue binary relations determined by *F *corresponding respectively to two sets of individual lineage profiles, (*R_1_*,...,*R_n_*) and $\left(R_{1}^{'},\ldots ,R_{n}^{'}\right)$. If for all pairs of alternatives *x *and *y *in a subset *S *of *X*, *xR_i_y *iff *xR'_i_y*, for all *i*, then *C*(*S, R*) and *C*(*S, R'*) are the same.

CONDITION 4 (non-dominance): There is no individual *i *such that for every element in the domain of *F*, for all *x, y *in *X*, if *xP_i_y *then *xPy*.

THEOREM: There is no LTM satisfying conditions C1, C2, C3 and C4.

The proof is prepared by two further definitions and a lemma.

DEFINITION 2. A set of lineages *V *is almost decisive for *x *against *y *if *xPy *whenever *xP_i_y *for every *i *in *V*, and *yP_i_x *for every *i *not in *V*.

DEFINITION 3. A set of lineages *V *is decisive for *x *against *y *if *xPy *when *xP_i_y *for every *i *in *V*.

A lineage *J *is picked out to denote *A*(*x, y*) to mean that *J *is almost decisive for *x *against *y*, and denote *D*(*x, y*) to mean that *J *is decisive for *x *against *y*. Note that *D*(*x, u*) implies *A*(*x, u*).

*Lemma*. If there is some individual *J *who is almost decisive for any ordered pair of alternatives, then a LTM satisfying conditions C1, C2, and C3 implies that *J *must be dominant.

*Proof*. Suppose that lineage *J *is almost decisive for some *x *against some *y*, i.e., there exists *x, y *in *X *such that *A*(*x*, *y*). Let *z *be another alternative, and let *i *refer to all lineages other than *J*. Assume *xP_J_y *and *yP_J_z*, and that *yP_i_x *and *yP_i_z*. Now, if *A*(*x*, *y*) and *xP_J_y *and *yP_i_x*, then *xPy*. Further, if *yP_J_z *and *yP_i_z*, then *yPz *from C2. But, if *xPy *and *yPz*, then *xPz *by the transitivity of the strict order relation *P*.

The result *xPz *is arrived at without any assumption about the propensity rankings of lineages other than *J *regarding *x *and *z*, although it is assumed that *yP_i_z *and *yP_i_x*. If these ranking w.r.t. *x *and *y*, and *y *and *z *have any effect on the tissue fate between *x *and *z*, we violate C3. Hence, *xPz *must be independent of these particular assumptions. Hence it must be the consequence of *xP_J_z *alone irrespective of the other orderings. But this means that *J *is decisive for *x *against *z*,

(1) if A(x,z), then D(x,z).

Now, suppose *zP_J_x *and *xP_J_y*, while *zP_i_x *and *yP_i_x*. By C2, we must have *zPx*. And since *A*(*x, y*) and *xP_J_y *and *yP_i_x*, we conclude that *xPy*. By transitivity, *zPy*. And this with only *zP_J_y*, without anything being specified about the rankings of the other lineages between *y *and *z*. Hence, *J *is decisive for *z *against *y*. The argument is analog to that used to arrive at (1),

(2) if A(x,z), then D(z,y).

Interchanging *y *and *z *in (2), we can similarly show that

(3) if A(x,z), then D(y,z).

By replacing *x *for *z*, *z *in place of *y*, and *y *in place of *x*, we obtain from (1),

(4) if A(y,z), then D(y,x).

Now,

if *A*(*x,y*), then *D*(*x,z*), from (1)

then *A*(*x,z*), from Definitions 2 and 3.

then *D*(*y,z*), from (3)

then *A*(*y,z*),

then *D*(*y,x*), from (4).

Therefore,

(5) if A(x,y), then D(y,x).

By interchanging *x *and *y *in (1), (2), and (5), we get

(6) if A(y,x), then D(y,z) and D(z,x) and D(x,y).

Next,

if *A*(*x,y*), then *D*(*y,x*), from (5)

then *A*(*y,x*).

Hence from (6), we have

(7) if A(x,y), then D(y,z) and D(z,x) and D(x,y).

Combining (1), (2), (5) and (7), one sees that *A*(*x, y*) implies that lineage *J *is decisive for every ordered pair of alternative fates from (*x, y, z*), given conditions C1, C2, C3. Thus *J *dominates over any set of three alternative fates containing *x *and *y*.

Considering now a larger set of alternatives, take any two alternatives *u *and *v *out of the entire set of alternatives. If *u *and *v *are so chosen that they are the same as *x *and *y*, then of course *D*(*u, v*) holds. If one of *u *and *v *is the same as one of *x *and *y*, say, *u *and *x *are the same but not *v *and *y*, then take the triple consisting of *x *(or *u*), *y *and *v*. Since *A*(*x, y*) holds, it again follows that *D*(*u, v*), and also *D*(*v, u*).

Finally, let both *u *and *v *be different from *x *and *y*. Now, first take (*x, y, u*), and we get *D*(*x, u*), which implies *A*(*x, u*). Next, take the triple (*x, u, v*). Since *A*(*x, u*), it follows from above that *D*(*u, v*), and also *D*(*v, u*). Thus *A*(*x, y*) for some *x *and *y*, implies *D*(*u, v*) for all possible ordered pairs (*u, v*). Therefore lineage *J *dominates, and the Lemma is proved.

In the final step the theorem is proved by using the Lemma.

*Proof*. It is shown that given conditions C1, C2, and C3, there must be a lineage which is almost decisive over some ordered pair of alternatives. We make the contrary supposition and show that it leads to an inconsistency.

For any pair of alternatives, there is at least one decisive set, the set of all lineages, as a consequence of C2. Thus, for every pair of alternatives there is also at least one almost decisive set, since a decisive set is also almost decisive. Compare all the sets of lineages that are almost decisive for some pair-wise choice, which is not necessarily the same pair, and from them choose the smalles one (or one of the smallest ones). Let this set be called *V*, and let it be almost decisive for *x *against *y*.

If *V *contains only one lineage, then we need not proceed further. If, however, it contains two or more lineages, we divide *V *into two parts, *V_1 _*containing a single individual, and *V_2 _*containing the rest of *V*. All individuals not contained in *V *form the set *V_3_*.

Due to C1, we can assume any logically possible combination of lineage rankings. We pick the following:

(1) For all *i *in *V_1_*, *xP_i_y *and *yP_i_z*.

(2) For all *j *in *V_2_*, *zP_j_x *and *xP_j_y*.

(3) For all *k *in *V_3_*, *yP*_*k*_*z *and *zP_k_x*.

Since *V *is almost decisive for *x *against *y*, and since every linage in *V *ranks *x *higher than *y*, and every lineage not in *V *does the opposite, we must have *xPy*. Between *y *and *z*, only *V_2 _*members rank *z *higher than *y*, and the rest *y *over z, so that if *zPy*, then *V_2 _*must be an almost decisive set. But *V *was chosen as the smallest almost decisive set, and *V_2 _*is smaller than that (being a proper subset of it). Hence *zPy *does not hold. Thus, for *R *to be complete as needed for condition C1, *yRz *must hold. But, if *xPy *and *yRz*, then *xPz*. But only the individual in *V_1 _*ranks *x *over *z*, the rest rank *z *over *x*, so that a certain lineage has turned out to be almost decisive. Hence there is a contradiction in the original supposition. Note, the proof works even if *V_3 _*is empty because as will be the case if *V *contains all the lineages, which has not been ruled out. The theorem now follows from the Lemma since a lineage almost decisive over some pair must be dominant. Q.E.D.

## Authors' contributions

Using Mathematical General Systems Theory, MDM provided rationale to adopt Arrow's impossibility theorem in life sciences. OW identified niche dominance as the focus of the study and developed the first draft of the manuscript. DS conducted the experiments that provided the basis for this investigation. All authors helped to write the following versions of the manuscript and approved it in its final version.
